# The Long and Winding Road: Uptake, Acceptability, and Potential Influencing Factors of COVID-19 Vaccination in Austria

**DOI:** 10.3390/vaccines9070790

**Published:** 2021-07-15

**Authors:** Isabel King, Petra Heidler, Roy Rillera Marzo

**Affiliations:** 1 Department of Public Health, St. Elizabeth University of Health and Social Work, 81106 Bratislava, Slovakia; iking@usc.edu.au; 2 Department of Exercise Physiology, School of Health and Behavioral Sciences, University of the Sunshine Coast, Sunshine Coast, QL 4558, Australia; 3 Department of International Business and Export Management, IMC University of Applied Sciences Krems, 3500 Krems an der Donau, Austria; 4 Department of Health Sciences, St. Pölten University of Applied Sciences, 3100 St. Pölten, Austria; 5 Department of Community Medicine, Faculty of Medicine, Asia Metropolitan University, Johor Bahru 81750, Malaysia; rrmtexas@yahoo.com; 6 Department of Community Medicine, International Medical School, Management and Science University, Shah Alam 40100, Malaysia

**Keywords:** COVID-19, vaccine, acceptance, vaccine hesitancy, public health, Austria

## Abstract

Acceptance of the COVID-19 vaccine will play a crucial role in combating the current pandemic. Vaccine rollouts have started in most countries. To reach the desirable vaccine coverage and to enhance its uptake, it is imperative to assess vaccine hesitancy. Methods: To assess the current vaccine acceptability in Austria and its influencing factors, an online survey was created and comprised fifteen questions segmented into a sociodemographic part and the acceptance and influencing factors of the approval of the COVID-19 vaccine. Results: In total, 70% of the 1350 respondents thought that the COVID-19 vaccine is an effective way to prevent and control the virus, while 13% disagreed and 17% were uncertain. Further, 71% approved the rapid development and rollout of the vaccine, while 55% were willing to accept the vaccine as soon as it became available, 18% did not want to get the vaccine, 17% wanted to delay, and 10% were already vaccinated. Conclusions: The results show a generally positive attitude towards the new COVID-19 vaccine. The doctor’s recommendation greatly influences the decision-making process, and tailored vaccine information can support a higher vaccine coverage.

## 1. Introduction

**Figure 1:**
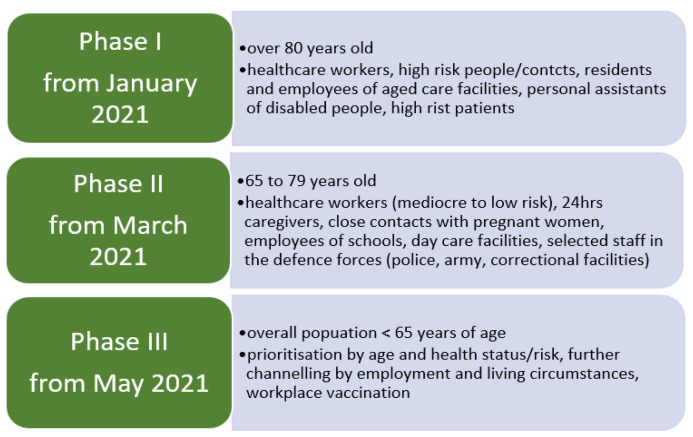
Austrian vaccination plan, displaying three phases from January 2021 until May 2021.

As of 8 April 2021, Austria had 568,916 registered cases of COVID-19 with 9586 deaths, while 525,682 people have fully recovered and 2516 people are currently hospitalized, with 578 being treated in intensive units. As of 29 March 2021, a total of 1,569,200 vaccine doses have been administered [1]. Austria was one of the first countries reporting COVID-19 cases, and complete lockdown measures were implemented in Austria for six weeks between 16 March and 25 April 2020, and again in December 2020 through to 8 February 2021. The current uptake in Austria stands at 20.96 doses of vaccine for every 100 people, which equals vaccination participation of 20% and ranks the country far behind states like Israel (117.72 doses/100 inhabitants), the United Kingdom (55.0), Hungary (38.49), and Morocco (22.89) [2]. As the situations have not shown significant progress, Austria is committed to developing an adequate vaccination strategy and course of action (see Figure 1) to improve its vaccination uptake to protect its population in the best way possible. However, it is not yet possible to define an ultimate procedure for distributing and administering the COVID-19 vaccination. Vaccinations are one of the most important, influential, and preventive measures in medicine, and significant medical success could be achieved through vaccinations and countries have developed free vaccination programs to enable access to vaccinations that are important for public health. Therefore, Austria has included the new corona vaccination in the regulation on recommended vaccinations in Austria [3].

Experience of disease outbreaks over the past two decades, like SARS and influenza in 2009, Ebola3 in 2014, and the Zika virus, has substantiated rapid progress towards vaccines for COVID-19 [4], and as it has become evident that an effective vaccine is required to bring the disease under control and prevent future outbreaks and global lockdowns. Moreover, introducing sensitive and robust disease severity measures encompassing the entire spectrum from mild to severe illness can support direct evidence-based diagnostic and therapeutic decisions [5]. Our focus on vaccine-related attitudes and intentions is essential because experts agree that having enough people vaccinate against COVID-19 is key to stemming the pandemic [6]. More broadly, negative attitudes towards vaccination in general and reduced vaccine uptake are an increasing public health concern and a seemingly emerging phenomenon, particularly in industrialized countries [7]. In that matter, public health authorities aim for global coverage of the COVID-19 vaccine; however, vaccine uptake and acceptability require the public to place their trust in global health as well as in the country’s response to the pandemic and positive sentiment towards the vaccine is crucial for high coverage and clear guidelines and standard operating procedures need to be established [5,8]. The growing number of communities and individuals refusing or delaying recommended vaccinations despite their availability poses a severe threat to global health. The World Health Organization (WHO) has identified vaccine hesitancy as one of the top ten global health threats in 2020, and global concerns are growing [9,10]. Vaccine hesitancy is a complex multifactorial problem with several determinants: vaccine efficacy, convenience, confidence, and trust in healthcare professionals. It is the main reason for low vaccination coverages [11,12,13]. Further, the accelerated pace of vaccine development has increased public anxiety and could adversely influence its acceptance [14].

Numerous studies have addressed vaccine hesitancy and its change in response to the COVID-19 pandemic [15]. There is only a little research on vaccine hesitancy in Austria to date, and further investigation into its contributing factors is necessary [16]. In Austria, physicians and family doctors are the most trusted source of health information, and their recommendations can substantially impact people’s attitudes towards vaccines [16]. This study aimed to understand better the factors contributing to vaccine reluctance in Austria to counteract and overcome obstacles that create uncertainty and skepticism and provide transparent and fact-based public health strategies to enhance immunization coverage. The objectives were to elicit the perceived efficacy of the new COVID-19 vaccine through research question 1: “Vaccination is a way of preventing and controlling COVID-19” (1), to elicit the acceptability and social approval of the rapid development of the new COVID-19 vaccine (2), to determine the willingness to get the COVID-19 vaccine (3), and to investigate influencing factors of COVID-19 vaccination acceptance among the Austrian population (4). We present findings from a survey of the likelihood of vaccine acceptance from a sample of 1350 respondents in Austria.

## 2. Methods

### 2.1. Survey Questionnaire and Participants

An anonymous cross-sectional study or transverse survey to assess the acceptability and factors influencing the attitude towards the COVID-19 vaccination in Austria was conducted. An online questionnaire in German was performed using the umfrageonline.com tool. The survey was modified from a questionnaire to be published in Malaysia. It comprised fifteen questions segmented into two parts, part A and part B. Part A contained nine questions about the sociodemographic profile of the participants, including age, gender, citizenship, marital status, annual household income, residential location setting (urban or rural), and educational level. Part B had six questions addressing acceptance, factors influencing the approval, and uptake of the COVID-19 vaccine. The link was distributed with an invitation to companies from various industries, e.g., the care sector, the business, the administration, the pedagogical sectors, non-profit organizations, nursing homes, and sports clubs.

Moreover, it was posted on social media and business platforms. A snowball sampling method was utilized. The survey was open from 18 February 2021 until 17 March 2021. The data collection began between Phase 1 and Phase 2 of the dissemination of the COVID-19 vaccination. Hence, our data span the early phase of the vaccination period and between lockdowns. The University Research Board approved the study. Participation in the survey was entirely voluntary, and participants gave their informed consent before answering the first question. No exclusion or inclusion criteria were defined, though the survey aimed to target people living in Austria to represent the country’s attitude. The primary measure of interest was the willingness of the participants to accept the vaccine as soon as it becomes available. Furthermore, the study focuses on the perceptions of the development of the COVID-19 vaccine and their vaccination attitudes in correlation with convenience and recommendations.

### 2.2. Data Analysis

The data obtained from the questionnaires were exported into Excel 2016, examined for errors, cleaned, shipped, and analyzed using IBM SPSS Statistics 27.0. Descriptive statistics were used as numbers and percentages to summarize the respondent’s sociodemographic characteristics. Cross-tabulations were performed to map the frequency distribution of selected variables in contingency tables to explore a potential relationship. A Chi-squared test was conducted to examine the significance of the association between demographics and factors influencing vaccine acceptance and determine whether there is a significant difference between expected frequencies and observed frequencies. A logistic regression model was employed on those variables that appeared to have a *p*-value < 0.05 in the multinominal analysis to identify determinants of participants’ acceptance of a COVID-19 vaccine. The significance of odds ratio (OR) in multivariate analyses was α = 0.05.

### 2.3. General Vaccine Hesitancy in Austria

Though data on vaccine hesitancy in Austria are limited, reviews of various papers and data reported by the WHO indicate a trend to increasing vaccine hesitancy and a declining child-vaccination rate [4,17,18,19]. For example, a survey on vaccine hesitancy in Austria, published in 2017, reported that, out of their 350 participants, 40 (11.4%) stated that they deliberately refused vaccinations, with fear of adverse effects, doubt of vaccine effectiveness, and distrust in the pharmaceutical industry being the main reasons for their reluctancy [17]. To give an example, in Austria, the vaccination coverage for measles and influence do not reach the recommended levels of vaccination by the WHO [17].

## 3. Results

### 3.1. Demographic Information

The number of respondents who gave their consent was 1395. Forty-five surveys were excluded owing to incomplete data. More than 80% of the participants were female and between 36 and 50 years old (43.15%), with a mean age of 44.99 years. There was little difference in the distribution of urban (58%) and rural (42%) residency among the respondent, with 94% being Austrian citizens. Further, 52% were tertiary educated and 37% were working full time. Almost 70% were married or in a solid partnership, and 36.3% of the respondents had a yearly family income between EUR 31,000 and 60,000. Table 1 presents the sociodemographic data of the 1350 valid responses.

### 3.2. Factors Impacting Acceptance of the COVID-19 Vaccine

Table 2 shows the acceptance, impact factors, and social approval of the COVID-19 vaccine. About 70% of all 1350 respondents thought that vaccination would be an effective way to prevent and control COVID-19, and 71% stated they trusted in science and research and approved the rapid approval of the vaccine. The ratio of the importance of convenience was almost evenly distributed between essential and not influential (49.6% and 50.4%, respectively). The doctor’s recommendation was a necessary factor for decision-making for 66% of the respondents, while the vaccine price was only essential to 23%. Further, 55% of the respondents would prefer to get the vaccine as soon as possible.

#### 3.2.1. Perceived Efficacy of the COVID-19 Vaccine

The survey aimed to elicit the perceived efficacy of a COVID-19 vaccine (“COVID-19 vaccination is an effective way to prevent and control COVID-19?”). Table 3 displays the cross-tabulation contingency table to investigate a potential correlation between education and the opinion on the COVID-19 vaccine being an effective way to prevent and control the disease.

The results of the Pearson correlation analysis with the correlation coefficient r = 27.11 at *p* ≤ 0.001 presented a statistically significant correlation between the level of education and perceived vaccine efficacy. Overall, 70.4% of the participants saw the COVID-19 vaccine as a protective measure to control and prevent the disease. People with tertiary education gave the highest count of affirmative answers. The numbers in uncertainties regarding the vaccine’s efficacy were higher than those of disapproval, at 13% and 16.5%, respectively. No significant correlation could be detected concerning age. People aged 66 or older tended to answer “no” rather than “uncertain”, at 9% and 5%, respectively, but 85.6% answered affirmative (Table 4 and Table 5).

#### 3.2.2. Doctor’s Recommendation and Vaccine Acceptance

This section explores the rates of vaccine acceptance in association with the doctor’s recommendation. Of all 1350 participants, 66.1% stated that their doctor’s vaccine recommendation was a crucial aspect in decision making, hypothesizing that the doctor’s recommendation for a COVID-19 vaccine has a more significant impact on older adults; the assumption can be accepted with a Pearson Chi-square value of 24.57 at *p* ≤ 0.001. The importance of the doctor’s recommendation was more substantial in people aged 66 years and older (84.6% within the age group) compared with people aged between 36 and 50, where 60.8% rated the doctor’s recommendation as necessary, and almost 40% said the opposite (Table 5). Regarding the doctor’s recommendation, 61.2% of those who valued the medical opinion of their doctor as paramount wanted to get the vaccine as soon as possible, 12.2% were already vaccinated, and only 9% would not want to get the vaccine (Table 6). A Pearson Chi-square value of 142.28 at *p* ≤ 0.001 suggests a statistically significant correlation between the doctor’s recommendation and the likelihood of vaccine acceptance.

Previous studies on vaccine hesitancy in Austria reported similar findings that the family general practitioners (GPs) enjoyed the highest levels of trust in their study population and noted that additional information communicated by GPs might be beneficial to counteract vaccine hesitancy [7,16].

#### 3.2.3. Likelihood to Get Vaccinated 

When asked if they would want to receive the COVID-19 vaccine as soon as possible, rather than wait, reject the vaccine, or already received the vaccine, 55.0% of the study participants stated that they would like to get the vaccine soon as possible, 17.7% reported rejecting the vaccine, while 10.2% were already vaccinated. Only 3.8% of people belonging to the age group of 66 plus were already vaccinated. In contrast, almost triple that number of people in the age groups 36–50 and 21–35 have received their vaccine, at 11.8% and 11.6%, respectively (see Table 7).

**Figure 2:**
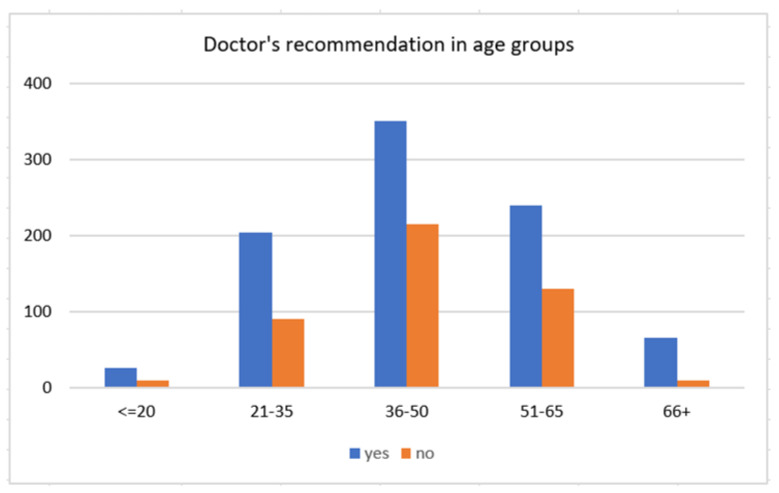
The reported importance of the doctor’s recommendation on decision making of whether to get the COVID-19 vaccine by age group.

Table 3 and Figure 2 show the distribution of the acceptance of the future COVID-19 vaccine between four categories (does not want the vaccine, wants the vaccine as soon as possible, undecided, already vaccinated) and its distribution between age groups. The majority (324) of the 743 respondents, who stated they would accept the vaccine as soon as possible, were 36–65 years old. Two hundred and thirty-nine reported they do not want to get the vaccine at all, showing that a reluctant attitude is higher among people in the age groups 51–66 years (18.3% within the age group) and lower in the age groups younger than 20 years (13.8% within the age group) and 66 plus (11.5%). Among adults aged younger than 20 years, 24.1% were still undecided, compared with 5.1% of adults aged 66 and older.

#### 3.2.4. Vaccine Acceptance and Educational Level

**Figure 3:**
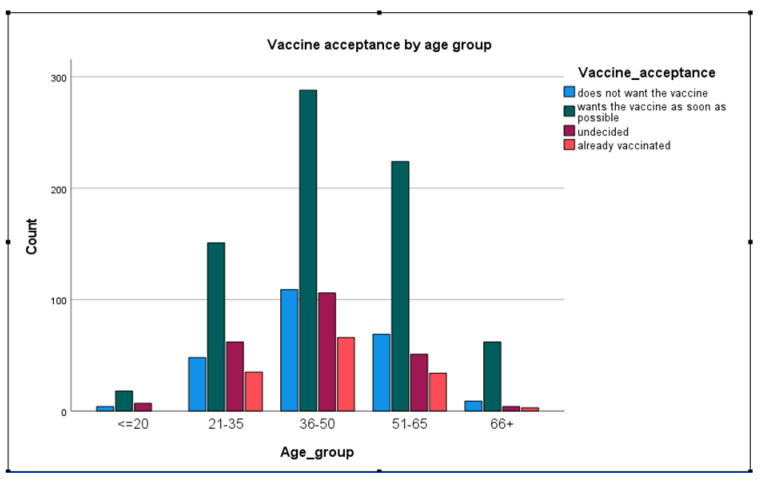
The willingness of the Austrian survey participants to receive the vaccination sooner or not at all in age groups.

**Figure 4:**
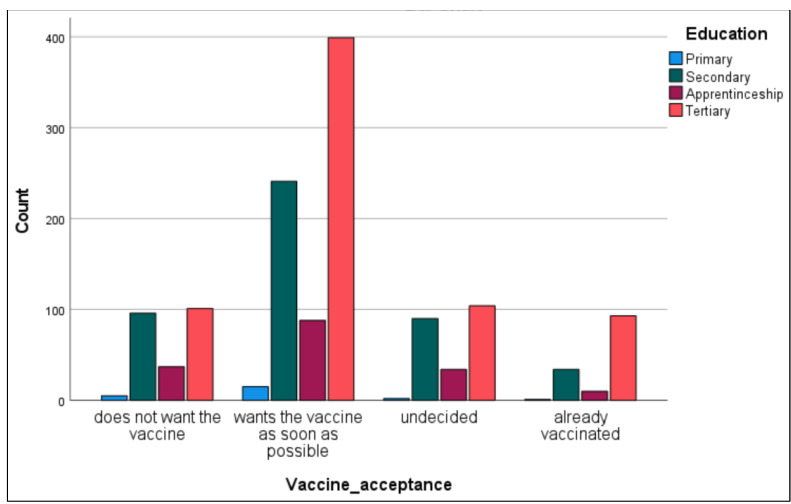
Acceptance of the COVID-19 vaccine in relation to the educational level in the Austrian participants.

Figure 3 and Figure 4, and Table 8 show the results regarding vaccine hesitancy in connection with the educational level of the survey participants and in general. Higher education was associated with higher acceptance of the COVID-19 vaccine. The results show that most people who wanted to accept the vaccine as soon as possible (53.7%) were tertiary educated, and 67.4% of those who already received their vaccine belonged to the higher education level. In multinominal logistic regression, significantly relevant outcomes show that participants with secondary education (OR 0.58, 95% CI 0.42–0.81) and aged 35–50 years old (OR 0.32, 95% CI 0.15–0.68) were less likely to accept the vaccine. No statistically significant effects on the likelihood of recommending vaccines were found for gender. A UK study from 2021 investigating predictors of COVID-19 vaccine hesitancy also reported a greater reluctance in people with a lower educational level [18].

#### 3.2.5. Vaccine Acceptance and Income

**Figure 5:**
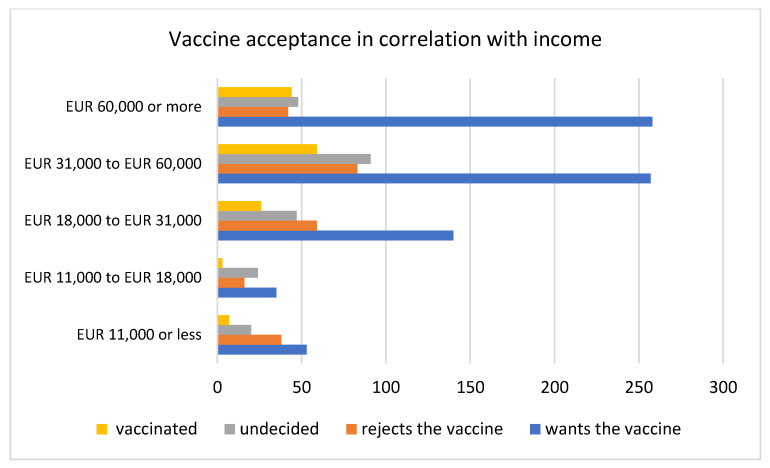
Vaccine acceptance in correlation with the yearly income of the survey participants in Austria.

Participants were asked to state their average income per household per year to see if an association between vaccine hesitancy and income can be identified. A Canadian study on determinants on vaccine hesitancy reported that, in the vaccine-hesitant group, people were more likely to have a lower education level and have a lower household income [20]. Our results in Table 9 and Figure 5 show the highest acceptance of the COVID-19 vaccine in the higher income group (60,000 Euros and more per year).

## 4. Discussion

Since the first diagnosed case of COVID-19 in December 2019, the number of new infections and fatalities in Austria has been increasing rapidly. National authorities are urged to respond to the pandemic appropriately. The World Health Organization (WHO) is yet to confirm and enhance treatment of the disease, which requires the administration of the newly developed vaccine. The rapid approval of the COVID-19 vaccine raises public concerns about its safety, and international surveys have suggested that general hesitancy to a COVID-19 vaccine has grown since its first release. The current willingness to accept a COVID-19 vaccine is insufficient to meet the requirements for community immunity [10]. Here, we report the findings of a brief survey addressing the attitude and openness towards the COVID-19 vaccine among Austrian citizens during stage one of the vaccination plan. The results showed that 23.4% of the people who already received their vaccine were aged between 35 and 50 years, estimating that those people were prioritized to be vaccinated owing to their professional background. Though the Austrian vaccination plan (Figure 1) aims to vaccinate older people and high-risk groups first, the ratio seems unbalanced. For a better understanding of these numbers, our survey could have included a question about the professional area of the participants. In this questionnaire study, a significantly lower response rate for male participants was obtained, which proposes the risk of gender-dependent errors in the analysis of the collected data—a reflection on a potential methodological bias has to be considered and improved in further studies. This apparent gender disparity results in the main limitation of this study. In Austria, insufficient vaccination promotion activity has been reported in the past, and stakeholders face complex challenges regarding the current events of the pandemic [16]. Furthermore, vaccination records are primarily paper-based and poorly standardized, if at all. Individuals moving from one region to another often face difficulties keeping vaccination records in place and up to date [21]. Although a national approach for vaccine administration is in place [3], greater effort has to be made towards promoting the new vaccine and must reach further than just supplying a free vaccine. Our results show that doctors significantly impact peoples’ decision making and the potential acceptance of the vaccine. Especially, older people might have higher trust in their physician owing to more frequent consultations for other health problems. Therefore, physicians should be made aware and work closely and synergetically with national health authorities in promoting vaccinations.

This study had several limitations, mainly selection biases. Firstly, the fact that 80% of the respondents were females raises several presumptions. Females may have been more inclined to agree to participate in our research, while males were more reluctant. Moreover, women have historically played a crucial role in the research and development of vaccinations. Women are more exposed to vaccinations and more involved in the vaccination status of their children [22,23]. Because of the small sample size of men (*n* = 241), the number is too small to test differences between men and women. Although the uneven distribution of men and women might be helpful to address the research questions, it may come at the cost of representativeness. Because the sampling method was snowball sampling, further studies should aim to recruit both genders and monitor the information while collecting closer or using a different sampling method. 

Another limitation, namely the selection bias regarding education, was recognized. This might have occurred because the link was distributed via e-mail to educational institutions and on social media.

In adults, tertiary education appeared to be correlated with a more positive attitude towards vaccination. People with primary education were more likely to be skeptical than people of more mature age. These findings are not entirely in line with other research on vaccine hesitancy and its impact factors. Several studies found that people with higher education were more likely to report vaccine hesitancy and identified higher education as a potential barrier to vaccine acceptance in specific settings [24,25,26], while in other findings, education was a significant factor in desire for COVID-19 vaccine [27,28]. COVID-19 vaccine acceptance increased with increasing age, income, and education level. These findings mirror the trends seen in other studies which surveyed the general population, for example, in the United States [29,30].

## 5. Conclusions

In Austria, studies on factors influencing vaccine hesitancy and acceptance are scarce. In our survey, vaccine hesitancy was low compared with media reports, criticizing a somewhat hesitant start of vaccinations in the country [27]. Our results suggest a generally positive attitude towards the new COVID-19 vaccine, with 70% of the participants showing their approval of the rapid development of the vaccine and more than half (55%) of the respondents being willing to take the vaccine soon as possible. At the time of the survey, 10% of the participants were already vaccinated. Adults with tertiary education (53.7%) and aged between 36 and 65 (68.9%) were most likely to get the vaccine. The doctor’s recommendation had a more significant influence on the decision-making process of older people, where 84.6% within the age group 66 and older reported to value the recommendation of their physician as high. Further, 61.2% of those who affirmed the importance of the doctor’s recommendation regarding the vaccine were willing to accept the vaccine as soon as possible. Primary physicians should be aware of their importance on people’s attitude towards a vaccine. More significant efforts are proposed to provide tailored vaccine information to aim for higher vaccine coverage.

## Figures and Tables

**Table 1:** Sociodemographic data, displaying age in categories, mean age, gender, and residential area of the 1350 valid responses.

Variables	Categories	Frequency	Percent
Total		1350	100
Age in category	≤20 years	29	2.1
Mean: 44.99	21 to 35	296	21.9
	36 to 50	569	42.1
	51 to 65	378	28.0
	66 or older	78	5.8
Gender	Female	1106	81.9
	Male	241	17.9
	x	3	0.2
Area of residency	Rural	564	41.8
	Urban	786	58.2
Citizenship	Austrian	1268	93.9
	Other	82	6.1
Educational level	Primary	23	1.7
	Apprenticeship	169	12.5
	Post-secondary education	461	34.1
	Tertiary education/University	697	51.6
Employment status	Full time	493	36.5
	Part-time	343	25.4
	Freelance/Self-employed	266	19.7
	Unemployed	38	2.8
	Student	70	5.2
	Retired	140	10.4
Marital status	Single	268	19.9
	Married/solid partnership	914	67.7
	Divorced	147	10.9
	Widowed	21	1.6
Yearly family income	EUR 11,000 or less	78	5.8
Mean:	EUR 11,000 to EUR 18,000	118	8.7
	EUR 18,000 to EUR 31,000	272	20.1
	EUR 31,000 to EUR 60,000	490	36.3
	EUR 60,000 or more	392	29.0

**Table 2:** Answers for the six questions of Part 2 of the survey examining acceptance, impact factors, and approval of the COVID-19 vaccine (*n* = 1350).

Items	N	%
COVID-19 vaccination is an effective way to prevent and control COVID-19		
yes	951	70.4
no	176	13.0
I am not sure	223	16.5
I trust in science and research and support the rapid approval of the COVID-19 vaccine and would like to accept it as soon as it is successfully developed and approved for listing		
yes	960	71.1
no	390	28.9
Vaccine convenience (vaccination method, frequency, distance to vaccination sites) is an important factor in vaccination decision-making		
yes	669	49.6
no	681	50.4
Doctor’s recommendation is an important factor in vaccination decision-making		
yes	892	66.1
no	458	33.9
Vaccine price is an important factor in vaccination decision-making		
yes	305	22.6
no	1045	77.4
I would accept the Coronavirus vaccine as soon as it becomes available		
Yes, the faster the better	743	55.0
I do not want to get the vaccine	239	17.7
I am not sure	230	17.0
I already got the vaccine	138	10.2

**Table 3:** Contingency table of the cross-tabulation between education and the belief of the COVID-19 vaccine being an effective way to prevent and control COVID-19 in absolute numbers and percentages.

Vaccine as an Effective Preventative Measure Education-Cross-Tabulation							
			Education				Total
			Primary	Secondary	Apprenticeship	Tertiary	
Vacine_protective_measure_	no	Count	4	70	28	74	176
		% within Vacine_efficacy	2.30%	39.80%	15.90%	42.00%	100.00%
		% within Education	17.40%	15.20%	16.60%	10.60%	13.00%
	yes	Count	16	299	103	533	951
		% within Vacine_efficacy	1.70%	31.40%	10.80%	56.00%	100.00%
		% within Education	69.60%	64.90%	60.90%	76.50%	70.40%
	uncertain	Count	3	92	38	90	223
		% within Vacine_efficacy	1.30%	41.30%	17.00%	40.40%	100.00%
		% within Education	13.00%	20.00%	22.50%	12.90%	16.50%
Total		Count	23	461	169	697	1350
		% within Vacine_efficacy	1.70%	34.10%	12.50%	51.60%	100.00%
		% within Education	100.00%	100.00%	100.00%	100.00%	100.00%

**Table 4:** Cross-tabulation of the division of the participants’ opinion on the COVID-19 vaccination being an effective way to prevent and control COVID-19 in absolute numbers and percentages by age groups.

Vaccine as an Effective Protective Measure Age_Group-Cross-Tabulation								
			Age_Group					Total
			≤20	21–35	36–50	51–65	66+	
Vaccine_protective measure	no	Count	1	33	87	48	7	176
		% within Vaccine efficacy	0.60%	18.80%	49.40%	27.30%	4.00%	100.00%
		% within Age_group	3.40%	11.10%	15.30%	12.70%	9.00%	13.00%
	yes	Count	23	217	381	263	67	951
		% within Vaccine efficacy	2.40%	22.80%	40.10%	27.70%	7.00%	100.00%
		% within Age_group	79.30%	73.30%	67.00%	69.60%	85.90%	70.40%
	uncertain	Count	5	46	101	67	4	223
		% within Vaccine efficacy	2.20%	20.60%	45.30%	30.00%	1.80%	100.00%
		% within Age_group	17.20%	15.50%	17.80%	17.70%	5.10%	16.50%
Total		Count	29	296	569	378	78	1350
		% within Vaccine efficacy	2.10%	21.90%	42.10%	28.00%	5.80%	100.00%
		% within Age_group	100.00%	100.00%	100.00%	100.00%	100.00%	100.00%

**Table 5:** Cross-tabulation of the importance of the doctor’s recommendation on the willingness to receive the COVID-19 vaccine by age group in total numbers and percentages.

Doctor’s Recommendation Age_Group Crosstabulation								
			Age_Group					Total
			≤20	21–35	36–50	51–65	66+	
Doctor´s_recommendation	no	Count	5	89	223	129	12	458
		% within DR	1.10%	19.40%	48.70%	28.20%	2.60%	100.00%
		% within Age_group	17.20%	30.10%	39.20%	34.10%	15.40%	33.90%
	yes	Count	24	207	346	249	66	892
		% within DR	2.70%	23.20%	38.80%	27.90%	7.40%	100.00%
		% within Age_group	82.80%	69.90%	60.80%	65.90%	84.60%	66.10%
Total		Count	29	296	569	378	78	1350
		% within DR	2.10%	21.90%	42.10%	28.00%	5.80%	100.00%
		% within Age_group	100.00%	100.00%	100.00%	100.00%	100.00%	100.00%

**Table 6:** Cross-tabulation of vaccine acceptance by doctor’s recommendation.

Vaccine Acceptance Doctor’s Recommendation-Cross-Tabulation					
			Doctor’s_Recommendation		Total
			no	yes	
Vaccine_acceptance	does not want the vaccine	Count	159	80	239
		% within VA	66.50%	33.50%	100.00%
		% within DR	34.70%	9.00%	17.70%
	wants the vaccine as soon as possible	Count	197	546	743
		% within VA	26.50%	73.50%	100.00%
		% within DR	43.00%	61.20%	55.00%
	undecided	Count	73	157	230
		% within VA	31.70%	68.30%	100.00%
		% within DR	15.90%	17.60%	17.00%
	already vaccinated	Count	29	109	138
		% within VA	21.00%	79.00%	100.00%
		% within DR	6.30%	12.20%	10.20%
Total		Count	458	892	1350
		% within VA	33.90%	66.10%	100.00%
		% within DR	100.00%	100.00%	100.00%

**Table 7:** Cross-tabulation of vaccine acceptance by age group.

Vaccine Acceptance by Age Group-Cross-Tabulation								
			Age_Group					Total
			≤20	21–35	36–50	51–65	66+	
Vaccine acceptance	does not want the vaccine	Count	4	48	109	69	9	239
		% within VA	1.70%	20.10%	45.60%	28.90%	3.80%	100.00%
		% within Age_group	13.80%	16.20%	19.20%	18.30%	11.50%	17.70%
	wants the vaccine as soon as possible	Count	18	151	288	224	62	743
		% within VA	2.40%	20.30%	38.80%	30.10%	8.30%	100.00%
		% within Age_group	62.10%	51.00%	50.60%	59.30%	79.50%	55.00%
	undecided	Count	7	62	106	51	4	230
		% within VA	3.00%	27.00%	46.10%	22.20%	1.70%	100.00%
		% within Age_group	24.10%	20.90%	18.60%	13.50%	5.10%	17.00%
	already vaccinated	Count	0	35	66	34	3	138
		% within VA	0.00%	25.40%	47.80%	24.60%	2.20%	100.00%
		% within Age_group	0.00%	11.80%	11.60%	9.00%	3.80%	10.20%
Total		Count	29	296	569	378	78	1350
		% within VA	2.10%	21.90%	42.10%	28.00%	5.80%	100.00%
		% within Age_group	100.00%	100.00%	100.00%	100.00%	100.00%	100.00%

**Table 8:** Vaccine acceptance by educational level of the respondents.

Vaccine Acceptance by Education-Cross-Tabulation							
			Education				Total
			Primary	Secondary	Apprenticeship	Tertiary	
Vaccine acceptance	does not want the vaccine	Count	5	96	37	101	239
		% within VA	2.10%	40.20%	15.50%	42.30%	100.00%
		% within Education	21.70%	20.80%	21.90%	14.50%	17.70%
	wants the vaccine as soon as possible	Count	15	241	88	399	743
		% within VA	2.00%	32.40%	11.80%	53.70%	100.00%
		% within Education	65.20%	52.30%	52.10%	57.20%	55.00%
	undecided	Count	2	90	34	104	230
		% within VA	0.90%	39.10%	14.80%	45.20%	100.00%
		% within Education	8.70%	19.50%	20.10%	14.90%	17.00%
	already vaccinated	Count	1	34	10	93	138
		% within VA	0.70%	24.60%	7.20%	67.40%	100.00%
		% within Education	4.30%	7.40%	5.90%	13.30%	10.20%
Total		Count	23	461	169	697	1350
		% within VA	1.70%	34.10%	12.50%	51.60%	100.00%
		% within Education	100.00%	100.00%	100.00%	100.00%	100.00%

**Table 9:** Acceptability of the COVID-19 vaccine among Austrian survey participants depending on income (per year in Euros).

Vaccine Acceptance Relating to Income							
			Does Not Want the Vaccine	Wants the Vaccine as Soon as Possible	Undecided	Already Vaccinated	Total
Income in EUR/year/houshold							
<11,000		Count	16	35	24	3	78
		% within Income	20.5%	44.9%	30.8%	3.8%	100.0%
		% within Vaccine_acceptance	6.7%	4.7%	10.4%	2.2%	5.8%
11–18,000		Count	38	53	20	7	118
		% within Income	32.2%	44.9%	16.9%	5.9%	100.0%
		% within Vaccine_acceptance	15.9%	7.1%	8.7%	5.1%	8.7%
18–31,000		Count	59	140	47	26	272
		% within Income	21.7%	51.5%	17.3%	9.6%	100.0%
		% within Vaccine_acceptance	24.7%	18.8%	20.4%	18.8%	20.1%
31–60,000		Count	83	257	91	59	490
		% within Income	16.9%	52.4%	18.6%	12.0%	100.0%
		% within Vaccine_acceptance	34.7%	34.6%	39.6%	42.8%	36.3%
60,000+		Count	43	258	48	43	392
		% within Income	11.0%	65.8%	12.2%	11.0%	100.0%
		% within Vaccine_acceptance	18.0%	34.7%	20.9%	31.2%	29.0%
Total		Count	239	743	230	138	1350
